# Prevalence of Suicidal Ideation and Its Association with Positive Affect in Working Women: A Day Reconstruction Study

**DOI:** 10.3389/fpsyg.2017.00285

**Published:** 2017-03-21

**Authors:** Lili Tian, Ying Yang, Huijing Yang, E. Scott Huebner

**Affiliations:** ^1^School of Psychology, South China Normal UniversityGuangzhou, China; ^2^Center for Studies of Psychological Application, South China Normal UniversityGuangzhou, China; ^3^Guangdong Key Laboratory of Mental Health and Cognitive Science, South China Normal UniversityGuangzhou, China; ^4^Department of Psychology, University of South CarolinaColumbia, SC, USA

**Keywords:** positive affect, affective instability, suicidal ideation, day reconstruction method, working women

## Abstract

The suicide rate for females in China is the second highest worldwide, and China is the only country in the world in which the rate of suicides is higher for women than men. Affective instability has been shown to be a strong predictor of suicidal ideation, particularly among women. However, prior research has mainly focused on the impact of women's negative affect on suicidal ideation, ignoring the influence of positive affect on suicidal ideation. Studies have revealed that hopelessness, which is 1.3 times more important than depression for explaining suicidal ideation, is driven more by low levels of positive affect than by high levels of negative affect. Although positive affect has also been found to be related to suicidal ideation, and it demonstrates independent, beneficial effects on mental health, much remains to be learned about the association between positive affective instability and suicidal ideation. Therefore, we investigated the prevalence of suicidal ideation among Chinese working women and explored the differences between working women with and without suicidal ideation in the intensity and daily variability of positive affect. A total of 222 young working women of ages 22–36 years (*M* = 27.64, *SD* = 3.73) were recruited from a free weekend psychology lecture. The women subsequently completed a daily diary Day Reconstruction Method (DRM) as well as a suicidal ideation questionnaire. We used hierarchical linear modeling (HLM) to analyze the data, and the results showed that: (1) 10.81% of participates reported suicidal ideation, the intensity of positive affect (happiness, warmth/friendliness, interest and relaxation/calmness) was significantly lower for women with suicidal ideation compared to women without suicidal ideation; (2) differing diurnal patterns of positive emotions were observed between women with and without suicidal ideation; women with suicidal ideation demonstrated a significantly lower trend of growth and a higher volatility in happiness, warmth/friendliness, relaxation/calmness. Given that lower intensity of positive affect and greater affective instability significantly predicted suicidal ideation in Chinese women, researchers should pay more attention to the role of positive affect in female suicide prevention research and intervention efforts in the future.

## Introduction

Previous research has revealed that suicide is the 10th leading cause of death worldwide (Hawton and van Heeringen, 2009). The WHO also estimated that 55% of suicides occur in individuals between the ages of 15–44 years (World Health Organization, 2012). Suicide in China accounts for approximately 20% of all suicides globally (Yang et al., 2013). Moreover, it is the highest leading cause of death for persons of ages 15–34 in China (Phillips et al., 2002a). In recent years, the phenomenon of suicide in the workplace has increased gradually (Mohseni-Cheraghlou, 2013). Numerous studies have found that certain occupations are at elevated risk of suicide compared with the general population, and females display a higher risk of suicide than males among the working population (Milner et al., 2016). Discrimination against global women significantly correlated with the prevalence of suicide among females and the high suicide ratio of females compared to males in Asia may in part be due to problems related specifically to family preferences for sons over daughters as revealed by the much higher intra-household investments in caring for, nurturing, and allocating resources to sons relative to daughters (Kwak et al., 2016; Pugh et al., 2016). One report estimated that the death rates by suicide of women in China ranked second highest in the world (Phillips et al., 2002a). Furthermore, China is the only country in which the suicide rate for women is higher than that for men (Phillips et al., 2002b).

Previous research on suicides among Chinese women has mostly focused on rural, unemployed women; little is known about the suicides of urban, working women. However, studies in China have found that working people (e.g., teachers) in urban areas report significant higher stress than working people in rural areas (Xu and Gao, 2003; Dong, 2014). Furthermore, urban women of childbearing age also report more stress than rural women of childbearing age (Xu and Ma, 2016). Moreover, research in other country has shown that employed women experience stress, anxiety and depression at higher frequencies than unemployed women (Anees and Khyrunnisa, 2015). Furthermore, the extant research indicates that suicide risk by occupational groups differs by gender, with the number of female suicides exceeding male suicides (Hawton et al., 2001; Sun and Ye, 2007). Thus, suicide among Chinese working women deserves more attention.

### The relationship between suicidal ideation and affect

Multiple, additional factors are implicated in risk for suicidal ideation, including distal risk factors, such as temperamental characteristics (e.g., impulsivity; Askenazy et al., 2003), cognitive factors (e.g., negative cognitive style; Stange et al., 2015), emotional competence (Kwok and Shek, 2010), and proximal risk factors, such as life events (Rew et al., 2016) and perceived stress (Cole et al., 2015). Moreover, prior studies have shown that affect has repeatedly been identified as a key predictor of suicidal ideation (Law et al., 2015). Individual experiences of different affective states have been linked to suicidal ideation, after controlling for other factors, such as personality (Liu, 2004). In addition, women are frequently reported to be more affectively sensitive than men (Thayer et al., 2003); thus, our research focused on the relations between affective states and suicidal ideation among women.

### The relationship between suicidal ideation and negative affect

Negative affect, including depression and anxiety, is strongly related to suicidal ideation (Isacsson, 2000; Guillaume et al., 2011). Women with depression report more suicidal ideation than men with depression (Brown et al., 2011), and the prediction of suicidal ideation from depression reports is more accurate among women than men (Stephenson et al., 2006). Thus, although the extant literature reveals robust evidence that negative affect and suicidal ideation and suicide are linked among women, a paucity of research has addressed the association between positive affect and suicidal ideation.

### The relationship between suicidal ideation and positive affect

Traditional mental health models have focused on negative affect, conceptualizing mental health as the absence of negative affect or disability. Nevertheless, research evidence is increasingly suggesting that positive affect has independent, beneficial effects on mental health (Dockray and Steptoe, 2010) and physical functioning (Garland et al., 2010; Koval et al., 2013). Studies have found that hopelessness, which is 1.3 times more important than depression for explaining suicidal ideation, is driven more by low levels of positive affect than by high levels of negative affect (Beck et al., 1993; Bryan et al., 2013). Similarly, in a 6-month prospective study of hospitalized adolescents, lower frequencies of positive affect were associated with shorter intervals in manifesting suicidal ideation, whereas negative affect was unrelated to manifesting suicidal ideation (Yen et al., 2013).

### The relationship between suicidal ideation and affective instability

Existing research has indicated that both negative affect and positive affect are associated with suicidal ideation. Moreover, evidence also suggests that instability in emotional experiences appears to play a critical role in suicidal ideation (Marwaha et al., 2014). A cross-cultural study involving six developing countries suggested that individual differences in the experience of affective instability was linked significantly to depression, which was the key predictor of suicidal ideation (Hawton et al., 2013). Additionally, affective instability was positively associated with suicidal behaviors (Fortson, 2004). Increases in affective instability could thus increase suicidal ideation through the repeated reactivation of latent suicidal cognitions (Palmier-Claus et al., 2012a). Similarly, individuals with higher levels of affective instability were more likely to experience suicidal ideation, perhaps because they did not utilize positive coping styles (e.g., seeking help) when facing major life events (Rudd, 2006). Although the extant literature supports the notion that higher levels of affective instability correlate with increased suicidal ideation, research has primarily focused on the impact of variability in negative affect (Palmier-Claus et al., 2012b), disregarding the potential impact of variability in positive affect on suicidal ideation. However, results from a number of studies suggest that positive affective instability, including both excesses and deficiencies in positive affect, shows consistent negative associations with mental health problems (Gruber, 2011). Compared with negative affective instability, positive affective instability has demonstrated a greater ability to predict individual psychological resilience (Cohn et al., 2009), unipolar depression (Geschwind et al., 2011) and bipolar disorder (Watson and Naragon-Gainey, 2010). However, little is known about the direct relationship between positive affective instability and suicidal ideation. Moreover, differences in hormonal variations between females and males may relate to the higher levels of affective instability reported among females (Wu et al., 2014). Perhaps relatedly, suicidal ideation for women appears to occur more frequently during the menstrual cycle (Smith et al., 2015).

### Measurement of affective instability

Multiple assessments of immediate, momentary emotions across 1 day or more are used to construct diurnal patterns. Greater numbers of assessments per day allow for the detection of finer-grained patterns. Recently, several studies of affect have been based on the experience sampling or ecological momentary assessment method (Stone and Shiffman, 1994; Hektner et al., 2007). The Day Reconstruction Method (DRM), a new procedure to assess affect, is similar to the Experience-Sampling Method (ESM) in its efforts to minimize retrospection biases, however, the DRM is less burdensome than the ESM. The first version of the DRM, which was employed with 909 working women in Texas, showed evidence of the practicability of both DRM and ESM (Stone et al., 2006). Recently, Dockray et al. (2010) used DRM affect ratings to compare to contemporaneous EMA ratings in a sample of 94 working women monitored over work and leisure days. The results indicated that the two methods produced very similar profiles of change over both work and leisure days. The average *intensity* of ratings was identical to the two methods for assessing *happiness* at almost all time points. The between-person correlations between methods were high after adjusting for attenuation, ranging from 0.58 to 0.90. DRM was structured to provide accurate and detailed retrieval of the objective circumstances of the previous day as well as individuals' affective experiences during the major activities of the day (Kahneman et al., 2004).

DRM had been widely used to capture the positive psychological variable, such as well-being (Tadić et al., 2013; Oerlemans and Bakker, 2014) and positive affect (Bhattacharyya et al., 2008; Daly et al., 2010). Given that DRM reports encompass 1 full day, DRM appears to be a tool well-suited for sampling across variety of different emotions and situations (Dockray et al., 2010). Furthermore, moment-based methods, like DRM, may be especially appropriate for studying the positive affect of Chinese persons, since Chinese people do not express their feelings directly based on Chinese traditional culture (e.g., Confucian culture; Stankov, 2010; Fang and Faure, 2011), and they display more expressive suppression (Simon et al., 2013) with the method of global retrospective reports (Chen and Chen, 2006). For these reasons, we used DRM to measure intensity of positive affect and affective instability of Chinese working women.

## The current study

Based on the aforementioned literature, we investigated the prevalence of suicidal ideation among working women and explored the differences between working women with and without suicidal ideation in the intensity and daily variability of positive affect. Specifically, we formulated two hypotheses: (1) participants will differ significantly on levels of positive affect, with women without suicidal ideation exhibiting higher positive affect intensity; and (2) participants will show significant differences in positive affect instability, with women with suicidal ideation exhibiting decreasing positive affect and greater variability in positive affect.

## Materials and methods

### Participants

A total of 231 women were recruited from a free weekend psychology lecture in the Guangdong province of China. All participants worked full-time in Guangdong and volunteered to complete the online questionnaire. With the exception of nine women who left early, the remaining 222 women completed the entire questionnaire. Thus, the 222 women who completed the questionnaire comprised the sample. All participants received written and oral information about the nature of this study (e.g., aim, methods, instruments, anonymity). The researchers answered all eventual questions. Written consent forms were collected from all participants, and all participation was voluntary as well as anonymous. The sample represented several industries, including sales, administration, education and hospitality. Of the 222 working women of ages 22–36 years (*M* = 27.64, *SD* = 3.73), 132 were unmarried (59.46 %); 32 were married without children (14.41%) and 58 were married with children (26.13%). A total of 144 had earned a bachelor's degree (64.86%) and 78 had earned a junior college degree (35.14%). Thirty-two women were experiencing menstruation on the day before participating in the research.

### Measures

#### Positive affect

Positive Affect was measured by the Computer-assisted Online DRM. Participants were asked to create a diary reconstructing the preceding day (“yesterday”) into a sequence of episodes. The episodes were restricted to a time-period of between 20 min and 2 h and were demarcated at the participants' discretion based on any significant changes during the day (e.g., change of place, activity, mood, or the presence of others).

Momentary positive affect was measured by a series of four affect items. The items were happy, warm/friendly, interested and relaxed/calm, all of which were adapted from prior research using the DRM in studies within women (Stone et al., 2006; Daly et al., 2010). Participants were asked to what extent they experienced a given affect using response options ranging from 0 (not at all) to 6 (very much) with higher scores reflecting higher intensity levels of each affect. For the purposes of this research, the indicator of each affect was the average level of each affect across all episodes. Hierarchical linear modeling (HLM) is generally preferable to analyze DRM data in that it allows for unequal numbers of repeated assessments, missing data, autocorrelation among repeated measures, and various error structures (Stone et al., 2006).

#### Suicidal ideation

Suicidal ideation was operationalized as the response to item 9 from the revised Beck Depression Inventory (BDI; Beck et al., 1979). Specifically, the question was “Have you had any thoughts or desire to commit suicide in the past month?” The response options were “0” (I haven't had any thoughts of suicide); “1” (I had suicidal thoughts, but I am not going to commit suicide); “2” (I want to commit suicide); “3” (I would commit suicide if there was a chance). Individuals who endorsed any thoughts of suicide (i.e., ratings of 1–3) were classified as positive for suicidal ideation. Prior research indicates that the suicidal ideation item on the BDI has adequate concurrent validity, as assessed by its correlation with other measures of current suicidal ideation, such as the clinician-administered Scale for Suicidal Ideation (*r* = 0.56; Beck et al., 1997), Beck Scale for Suicide Ideation (*r* = 0.69; Beck et al., 1979; Steer et al., 1993) and the self-report Beck Scale for Suicidal Ideation (*r* = 0.69; Beck et al., 1988), among outpatient samples.

### Procedure

Every participant received information describing our research and an informed consent form. The study was approved by School of Psychology Research Ethics Committee of South China Normal University. The participants completed an on-line questionnaire, which included demographic information, the DRM and the measure of suicidal ideation. All participants were compensated with an honorarium.

### Data analysis

Preliminary analyses including, descriptive statistics, and *t*-tests, were conducted using the SPSS 16.0 statistical package. Subsequent data analyses were performed using HLM (hierarchical linear models) version 6.08.

For the multilevel analysis, we used each episode's midpoint (the average of beginning and ending times) to represent when the episode occurred. These midpoints were categorized into 14 (from 7:00 a.m. to 21:00 p.m.) 1-h blocks (e.g., 9:00–9:59 a.m.). The procedure has the advantage that the degrees of freedom for the inferential statistics are based appropriately on the number of episodes reported.

HLM was used to examine diurnal rhythms for each affect. First, we estimated the null model to test if the data were suitable for hierarchical linear modeling. Second, we estimated the linear growth model and non-linear growth model to test if there were diurnal rhythms for each affect with time as the level-1 independent variable. Third, we estimated the full model with time as a level-1 independent variable and suicidal ideation as a level-2 independent variable, and marital status and menstruation in level-2 as control variables to test if suicidal ideation related significantly to intercepts and slopes associated with diurnal rhythms for each affect.

## Results

### Descriptive statistics

As previously mentioned, suicidal ideation was defined as participants' responses to one item from the revised BDI. A total of 198 participants (89.19%) chose “0” and 24 participants (10.81%) chose “1,” and no one chose “2” or “3.” In this research, the detection rate of suicidal ideation within the past month was 10.81%. The mean episode length was 76 min, and the number of episodes of all the participants was 1857 (*M* = 8.36) among which 254 episodes (13.67%) were derived from suicide ideators.

### Differences between suicide ideators and non-suicide ideators on positive affects

The mean levels (across all respondents and episodes) for the positive affects, as measured by the DRM, are shown in Table 1. The results of the Independent sample *t*-tests revealed that non-suicide ideators reported significantly higher intensity of affect than suicide ideators.

**Table 1:** **Differences between positive affects for suicide ideators (*N* = 24) and nonsuicide ideators (*N* = 198)**.

**Positive affects**	**Non-suicide ideator *N* (episode) = 1603**	**Suicide ideator *N* (episode) = 254**	***t***
Happy	3.00 ± 2.07	1.94 ± 2.00	7.57^***^
Warm/friendly	2.87 ± 2.07	1.65 ± 1.94	8.80^***^
Interested	2.57 ± 2.02	1.67 ± 1.94	6.62^***^
Relaxed/calm	3.00 ± 1.95	1.80 ± 1.91	9.18^***^

*** *p < 0.001*.

### Comparison of diurnal rhythms of positive affects for suicide ideators and non-suicide ideators

#### Tests of the hierarchical linear modeling

Table 2 shows the results of chi-square tests for the four positive affects (all *p* < 0.001). The intra-class correlations of a two-level HLM null model (M0) showed that 30.2–42.0% of the total variance for the four affects resided at the within-person level, indicating that affects fluctuated substantially on a within-person level. Hence, Hierarchical Linear Modeling was fit for the data.

**Table 2:** **Tests of HLM of positive affects**.

**Positive affect**	**σ^2^**	**χ^2^**	***df***	**ICC**	**Deviance**
Happy	1.50	1203.37^***^	221	0.344	7585.73
Warm/friendly	1.84	1572.97^***^	221	0.420	7422.40
Interested	1.24	1021.96^***^	221	0.302	7565.01
Relaxed/calm	1.26	1145.72^***^	221	0.325	7411.24

ICC, Intra-class correlations; Deviance, Statistics for current covariance components model;

*** *p < 0.001*.

#### Tests of diurnal rhythm for each affect

Considering the frequent measurement and complex daily rhythm of affect, we examined both the linear and nonlinear components of time for each affect. First, we examined the linear model of affect as a function of time-varying predictor and established M1, Level 1: *y* = β0 + β1 (time) + *r;* Level 2: β0 = γ00 + μ0, β1 = γ10 + μ1. As shown in Table 3, the final estimation of fixed effects showed the intercept and slope were both significant for the four kinds of positive affects. Namely, a significant linear growth trend over time was observed for each affect. However, the estimation of random effects showed no significant differences among individuals for “warm/friendly” (χ^2^ warm/friendly = 229.19, *p* warm/friendly > 0.05).

**Table 3:** **Tests of diurnal rhythm for each affect**.

		**M**_**1**_				**M**_**2**_			
		**Estimate**	***t***	**χ^2^**	**ΔDeviance**	**Estimate**	***t***	**χ^2^**	**ΔDeviance**
Happy	Intercept	2.30	19.61^***^	654.47^***^	86.89^***^	2.15	16.14^***^	431.58^***^	82.09^***^
	Slope1	0.09	7.94^***^	317.59^***^		0.17	5.20^***^	173.96	
	Slope2	–				−0.00	−2.77^**^	166.57	
Warm/friendly	Intercept	2.52	23.04^***^	580.94^***^	9.84^**^	2.37	20.00^***^	371.29^***^	13.06^**^
	Slope1	0.03	3.80^***^	229.19		0.11	3.39^***^	225.58	
	Slope2	–				−0.01	−2.55^*^	199.43	
Interested	Intercept	1.88	17.64^***^	495.26^***^	106.04^***^	1.80	14.76^***^	384.37^***^	101.83^***^
	Slope1	0.10	9.20^***^	283.44^**^		0.14	4.05^***^	241.42	
	Slope2	–				−0.00	−1.40	206.37	
Relaxed/calm	Intercept	2.38	24.24^***^	460.86^***^	64.29^***^	2.30	20.29^***^	342.50^***^	56.89^***^
	Slope1	0.07	7.56^***^	269.00^**^		0.12	3.45^***^	226.54	
	Slope2	–				−0.00	−1.35	206.87	

*Deviance = difference compared to the null model*.

** p < 0.01,

*** *p < 0.001*.

We examined the nonlinear model of positive affects as a quadratic function of time and estimated M2, Level 1: *y* = β0 + β1 (time) + β2 (time)2 + *r*; Level 2: β0 = γ00 + μ0, β1 = γ10 + μ1, β2 = γ20 + μ2. As shown in Table 3, the linear model fit the data significantly better than the nonlinear model according to the reduced deviance compared with the null model. However, only the slope of “warm/friendly” was significant (*t* warm/friendly = −2.55, *p* warm/friendly < 0.05).

As for “warm/friendly,” both the hierarchical linear model and hierarchical nonlinear model fit the data better than the null model. Also, compared with the hierarchical linear model, the number of estimated parameters for the hierarchical nonlinear model increased by 3, while the deviance reduced 3.22 (*p* > 0.05). The improvement in the accuracy was obtained by increasing the order of the polynomial; therefore, the hierarchical nonlinear model was determined to be the better model, and the daily rhythm of “warm/friendly” was determined to be nonlinear. No statistically significant differences among individuals for “warm/friendly” were revealed by tests based on random effects (χ^2^ slope 1 = 225.58, *p* slope 1 > 0.05; χ^2^ slope 2 = 199.43, *p* slope 2 > 0.05).

#### Tests of the full model for each affect

Based on the tests of diurnal rhythm for each affect, we examined whether suicidal ideation was predictive of diurnal rhythms of positive affect levels. A two-level model M3 was estimated on the basis of the linear model M1 for “happy,” “interested,” “relaxed/calm” with suicidal ideation as the predictor in level 2. We controlled marital status and menstruation in level 2 for M3, which may have an influence on affect. Level 1: *y* = β0 + β1 (time) +*r*; level 2: β0 = γ00 + γ01 (suicidal ideation) + γ02 (marital status) + γ03 (menstruation) + μ0, β1 = γ10 + γ11 (suicidal ideation) + γ12 (marital status) + γ13 (menstruation) + μ1.

A two-level model M4 was estimated on the basis of a nonlinear model M2 for “warm/friendly” in the same way as M3. Level 1: *y* = β0 + β1 (time) + β2 (time)2 +*r*; level 2: β0 = γ00 + γ01 (suicidal ideation) + γ02 (marital status) + γ03 (menstruation) + μ0, β1 = γ10 + γ11 (suicidal ideation) + γ12 (marital status) + γ13 (menstruation) + μ1, β2 = γ20 + γ21 (suicidal ideation) + γ22 (marital status) + γ23 (menstruation) + μ2.

**Figure 1:**
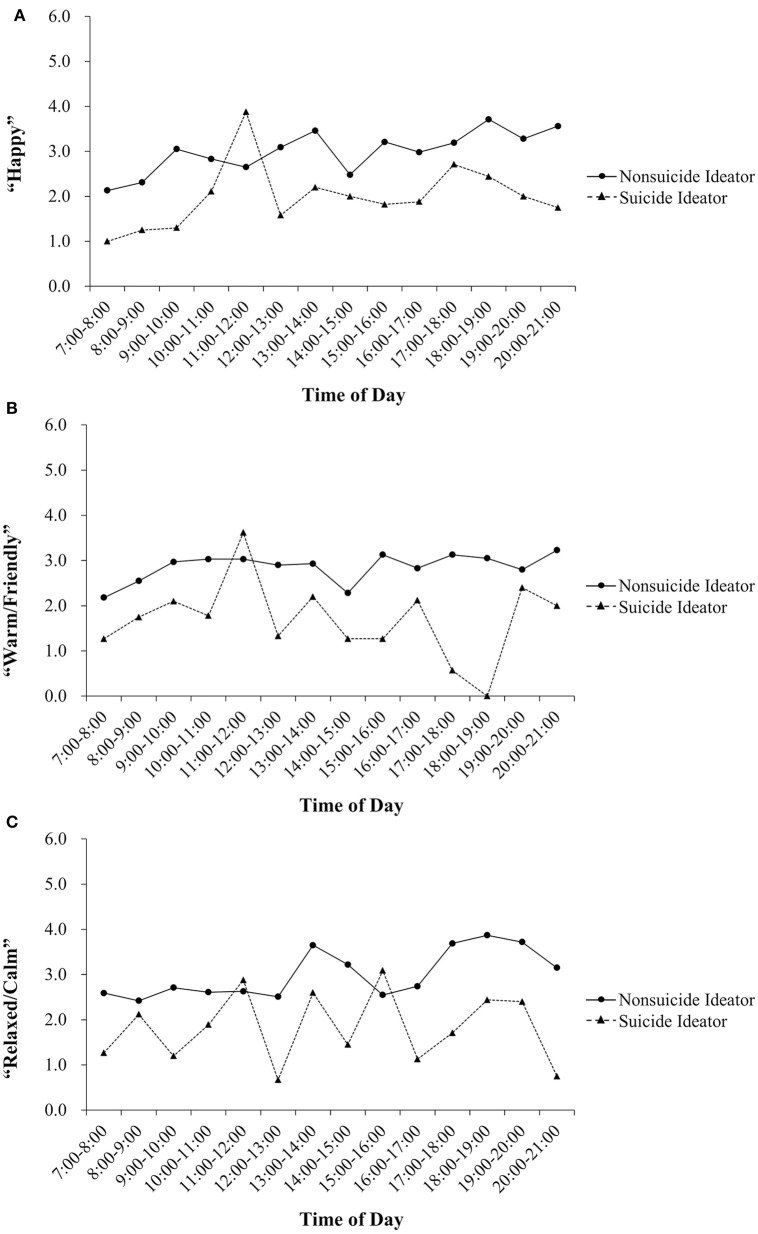
**Comparison of diurnal patterns of happy (A)**, warm/friendly **(B)** and relaxed/calm **(C)** for suicide ideators and nonsuicide ideators. Points are mean scores computed across hourly averages within each sample.

The results showed that under the condition of controlling marital status and menstruation, suicidal ideation was a significant predictor of the intercept of the “happy” daily rhythms, but not a significant predictor of the slope (see Table 4). An examination of the intercepts for the “happy” daily rhythm revealed that scores for the suicidal ideation group were significantly higher than the non-suicidal ideation group. With regard to the “warm/friendly” daily rhythms, suicidal ideation was a significant predictor of the two slopes, but not the intercept. For the “warm/friendly” daily rhythms, the suicidal ideation group showed lower intensity of affect from 7:00 a.m. to 21:00 p.m. With regard to the “relaxed/calm” daily rhythms, suicidal ideation significantly predicted the intercept and slope. In the “relaxed/calm” daily rhythm, the slope was higher and the level was lower for the suicidal ideation group. Suicidal ideation did not significantly predict the daily rhythms for the affect of “interested.” In addition, the daily rhythms of “happy,” “warm/friendly,” and “relaxed/calm” for the suicidal ideation group demonstrated more fluctuations than those for the daily rhythms of the non-suicidal ideation group (see Figure 1).

**Table 4:** **Tests of the full model for each affect**.

		**Variable**	**Estimate**	***SE***	***t***
Happy	Intercept	Intercept (γ_00_)	2.06	0.15	13.89^***^
		Suicidal ideation (γ_01_)	−0.76	0.30	−2.51^*^
		Marital status (γ_02_)	−0.26	0.14	1.84
		Menstruation (γ_03_)	1.09	0.39	2.78^**^
	Slope	Intercept (γ_10_)	0.09	0.01	6.81^***^
		Suicidal ideation (γ_11_)	−0.04	0.04	−0.98
		Marital status (γ_12_)	0.04	0.01	3.07^**^
		Menstruation (γ_13_)	−0.12	0.02	−5.02
Warm/friendly	Intercept	Intercept (γ_00_)	1.82	0.14	12.68^***^
		Suicidal ideation (γ_01_)	−0.34	0.36	−0.95
		Marital status (γ_02_)	0.58	0.12	4.60^***^
		Menstruation (γ_03_)	1.22	0.34	3.62^***^
	Slope 1	Intercept (γ_10_)	0.19	0.04	4.77^***^
		Suicidal ideation (γ_11_)	−0.29	0.08	−3.61^***^
		Marital status (γ_12_)	0.05	0.05	1.11
		Menstruation (γ_13_)	−0.44	0.07	−5.95^***^
	Slope 2	Intercept (γ_20_)	−0.01	0.00	−3.32^***^
		Suicidal ideation (γ_21_)	0.01	0.01	2.68^**^
		Marital status (γ_22_)	0.00	0.00	−1.05
		Menstruation (γ_23_)	0.02	0.01	4.06^***^
Interested	Intercept	Intercept (γ_00_)	1.56	0.12	12.68^***^
		Suicidal ideation (γ_01_)	−0.53	0.32	−1.64
		Marital status (γ_02_)	0.39	0.13	2.93^**^
		Menstruation (γ_03_)	0.81	0.28	2.86^**^
	Slope	Intercept (γ_10_)	0.10	0.01	9.03^***^
		Suicidal ideation (γ_11_)	−0.05	0.04	−1.24
		Marital status (γ_12_)	0.01	0.01	0.71
		Menstruation (γ_13_)	−0.04	0.03	−1.49
Relaxed/calm	Intercept	Intercept (γ_00_)	2.21	0.12	17.78^***^
		Suicidal ideation (γ_01_)	−0.66	0.29	−2.25^*^
		Marital status (γ_02_)	0.08	0.11	0.67
		Menstruation (γ_03_)	1.36	0.27	5.03^***^
	Slope	Intercept (γ_10_)	0.06	0.01	5.34^***^
		Suicidal ideation (γ_11_)	−0.07	0.03	−2.00^*^
		Marital status (γ_12_)	0.05	0.01	5.28^***^
		Menstruation (γ_13_)	−0.10	0.02	−4.39^***^

* p < 0.1,

** p < 0.01,

*** *p < 0.001*.

## Discussion

Globally, the prevalence rate for suicidal ideation is approximately 9.2% (Nock et al., 2008). Our result revealed a suicidal ideation rate of 10.81%, which is similar to the global suicide prevalence rate and the findings from other studies (around 10–14%, Mackenzie et al., 2011; Salve et al., 2012). In particular, female suicidal ideation rates assessed in Japan and Europe are 9.79 and 12% (Sugawara et al., 2012; Mandelli et al., 2015), respectively, (Mandelli et al., 2015), which are also comparable to the results in this study.

### Differences in positive affect between working women with and without suicidal ideation

The first aim of the research was to examine the difference in positive affect between working women with and without suicidal ideation (i.e., hypothesis 1). Results indicated that the positive affective intensity (i.e., happiness, warmth/friendliness, interest and relaxation/calmness) of women with suicidal ideation was significantly lower than that of women without suicidal ideation. Prior research has indicated that a lack of positive affect is associated with more mental health problems (Dockray and Steptoe, 2010), and problems of mental health are related to suicidal ideation (Cederbaum et al., 2014). Our finding was also consistent with other research in which people with suicidal ideation showed significantly less positive affect but more negative affect (e.g., anger, hostility, and guilt; Seidlitz et al., 2001). Moreover, the relative lack of positive affect was significantly associated with suicidal ideation as well as suicidal behavior even after controlling for a diagnosis of depression (Nock and Kazdin, 2002). Thus, our finding is in accordance with prior studies suggesting that the relative lack of positive affect is a predictive risk factor for suicidal ideation.

### Differences in positive affective instability between women with and without suicidal ideation

It is notable that we found a diurnal cycle of affect in working women, which is consistent with research by Stone et al. (2006). Stone used DRM to assess affect from 909 women over a working day, with the results showing that diurnal cycles were observed for most of the positive affects (e.g., happy, warm and enjoy). Affect changed in response to the stimulation from the environment. Nevertheless, across time, variations in affect showed some stability over the longer term. Furthermore, variations in affect in diurnal cycles may be influenced by the same diurnal endogenous factors (e.g., cortisol, growth hormone) and exogenous factors (e.g., working day) (Kivlighan et al., 2008).

Regarding our hypothesis 2, results supported our hypothesis that participants would show differences in positive affect instability, with women reporting suicidal ideation exhibiting decreasing positive affect. It is notable that we used the affect level of participants at 7 a.m. to analyze the intercept of linear growth and it reflected the range ability of affect in 1 day, since research has found that the peak of female affective instability in a day appears at that time (Stone et al., 2006). Under this condition, the slope of linear growth showed the variability and tendency of affective instability. The results indicated that for happiness, women with suicidal ideation demonstrated a lower range ability, after controlling for marital status and menstrual conditions. This result was also in accordance with previous research. The experience of happiness may help people have more confidence in the future and more quickly return to the normal range after negative life events, resulting in lower rates of suicidal ideation (Taylor and Armor, 1996). Thus, people with suicidal ideation should report less happiness. In addition, our finding demonstrated that women with suicidal ideation demonstrated a significantly lower variability for the affect of warmth/friendliness. Relationships with others likely worsen when women can not feel warm/kind, which may decrease their social support and lead to more suicidal ideation. Such a possibility is suggested by a recent study, which reported that participants who received home visits by commissioned welfare volunteers felt greater warmth and reported less risk of suicidal ideation (Noguchi et al., 2014). We also found that compared to women without suicidal ideation, those women with suicidal ideation displayed a significant variability for the affect of relaxation. Less relaxation has been significantly associated with more stress and more cortisol, and people with higher cortisol as well as stress have reported more suicidal ideation (Giletta et al., 2015), which is in accordance with our finding that the women with suicidal ideation reported lower levels of relaxation.

In general, there were significant differences for women with and without suicidal ideation in terms of both affect intensity and affective instability. As can be seen in Figure 1, the volatility of the affects of happiness, warm/kind and relaxation was higher in the women with suicidal ideation; this fits with our hypotheses 2 that women with suicidal ideation would exhibit greater volatility in affect. Based on the Dynamic Equilibrium Theory, happiness levels remained stable over time despite changes in most life circumstances, even major events (Headey, 2008). The stability of positive affect appears to plays an important role in various mental health conditions (Marwaha et al., 2013). For instance, affective instability has been related to diagnoses of depression and borderline personality disorder (Reich et al., 2014). Furthermore, affective instability has been shown to be a strong predictor of suicidal ideation (Yen et al., 2004), which is in accordance with our findings. Particularly, the variables of timing of menstrual periods and marital status played a role in the relation between suicide ideation and positive affective instability. Prior research has shown that participants report more suicide attempts during the menstrual period (Wu et al., 2014), and women experiencing menstruation are more likely to report affective instability (Romans et al., 2012). Additionally, marital status has been found to be strongly related to suicidal ideation (Tran Thi Thanh et al., 2006). Our results were consistent with previous research in that there were differences for women with or without suicidal ideation in positive affect instability after controlling for menstruation and marital status. The findings thus provided additional support for further study of the mechanisms that account for the relationship between suicidal ideation and positive affective instability among women.

## Limitations and strengths

Our study was limited by several factors. First, we lacked information regarding the careers of the working women and the comparison with other women without work. The limitation of the lack of this knowledge was exemplified by the higher risk of suicide among women (but not men) medical doctors compared to the general population (Hawton et al., 2001). This methodological shortcoming likely limited our ability to detect differences in characteristics of positive affect and suicidal ideation among working women. Second, this study did not take negative affect into account. Nevertheless, a substantial number of previous studies have consistently demonstrated a significant association between negative affect and suicidal ideation. Given that previous research has also shown that positive affect (but not negative affect) varies according to a sinusoidal 24-h rhythm centered around participants' average wake times (Hasler et al., 2008), we focused on the relationship between positive affect and suicidal ideation. Third, although one-item questionnaires have been widely used to assess suicidal ideation across Asia, such as China (Sun et al., 2017), Japan (Sugawara et al., 2012) and Korea (Yoo et al., 2016), the limitations of one-item measures (e.g., internal consistency reliability estimation, validity) should nevertheless be recognized. Fourth, menstruation is related to affect instability not just during the day it occurs but also some days before (Baca-Garcia et al., 2010); however, the questions about menses in our study were limited to the day when DRM was applied. Fifth, the sample was relatively homogeneous (e.g., young women from a specific region of China taking a psychology course who volunteered to complete the on-line survey). Future studies with more heterogeneous samples would enhance the ability to generalize from the findings. Finally, our study was based on cross-sectional data, which constrains claims of causality. Future studies should test the longitudinal associations between both positive and negative affect with suicidal ideation among Chinese working women in relation to Chinese non-working women.

Nevertheless, our research displayed a number of strengths. Firstly, nearly 20% of all suicides worldwide occur in China, and China is the only country in which the suicide rate for women is higher than that for men. We provides some of the first information regarding the characteristics of positive affective instability and its relation with suicidal ideation among Chinese working women whose suicide rate ranks among the top two nations in the world. Secondly, we used the method of DRM to assess affective instability and verified the daily rhythm of positive affect instability among women, which is consistent with the study of Kahneman et al. (2004). Finally, our research was the first to our knowledge to focus on the association between positive affect and suicidal ideation, with previous research focusing exclusively on the relation between negative affect and suicide. Moreover, we assessed the role of positive affect instability in relation to suicidal ideation. Notably, affective instability appears to be the strongest predictor of suicidal behavior, exceeding the amount of variance accounted for by negative affect (Marwaha et al., 2014).

## Implications

The results of our study underscore the importance of consideration of the relatively neglected roles of positive emotions in understanding suicidal ideation among working women. Specifically, our findings revealed that working women with suicidal ideation showed higher positive affect instability, after controlling for marital status and timing of the menstrual cycle. Thus, working women, particularly working women with positive affect instability and who were single, divorce, widowed or experiencing their menstrual period, represent a relatively high-risk group for suicide. Moreover, the World Health Organization needs to pay greater attention to this particular population. In addition, along with negative affect, positive affect and affective instability should be taken into consideration when assessing the suicidal risks for women. Such information should enable organizations to develop more systematic, comprehensive interventions to increase the positive affect of the women at risk and decrease their affective instability in order to protect them against suicide.

## Author contributions

All the authors (LT, YY, HY, and EH) substantially contributed to the conception and the design of the work. LT, YY, and HY participated to the acquisition of data. The two authors (LT and HY) analyzed and interpreted the data. The first author (LT) prepared the draft and the contributing authors (YY, HY, and EH) reviewed it critically and gave important intellectual content. All the authors (LT, YY, HY, and EH) worked for the final approval of the version to be published. All the authors (LT, YY, HY, and EH) are accountable for all the aspects of the work in ensuring that questions related to the accuracy or integrity of any part of the work are appropriately investigated and resolved.

### Conflict of interest statement

The authors declare that the research was conducted in the absence of any commercial or financial relationships that could be construed as a potential conflict of interest.
